# Denarcotized Opium in Fever

**Published:** 1855-03

**Authors:** W. S. Sinn

**Affiliations:** Ill.


### SIMPLE INTERMITTENT AND REMITTENT FEVER TREATED BY PARTIALLY DENARCOTIZED OPIUM.

BY W. S. SINN, M. D., ILL.

By some, opium is highly extolled in periodical fevers for meliorating the cold stage; others have thought it operated most beneficially in the interim or the remission, and a few solitary cases have been trusted to that alone during the whole period of the disease. Perhaps there is no disease that is as amenable to treatment, and its management so clearly pointed out, as that of paludal fever, that has been the subject of such various and widely diversified treatment. Every one knows that quinine is certainly and suddenly destructive to its progress, but something cheaper and newer is the ambition that inspires almost every one that is often called to treat it; for in districts where it prevails extensively, a small fortune in a short time is requisite to cover the expense of the prodigal dispenser of that drug.

The fact that led my mind to the foregoing treatment more particularly was, that when opium was given in combination with the cinchonic salts, that a less quantity of the latter was sufficient to defeat analogous cases in a shorter length of time, and in a manner I do not say *better*, but more satisfactory to myself and patients.

The manner in which I proceeded in the treatment of the aforementioned cases, was by cleaning the bowels with six grains of calomel, twenty to thirty grains of rhubarb, succeeding their administration in three doses two hours apart, with a small tablespoonful of castor oil, and beginning as soon as the bowels were well freed of their superabundance of fecal matter, if no gastric distress was present, with two grains of ipecac, and from three to eight grains of partially denarcotized opium every six hours, no matter at what stage of the disease. If there was irritation in the primæ viæ, the ipecac. was witheld and the opium given solitary.

In every case sleep soon overcame the patient, the pains in the head and back as well as limbs were soon dissipated, copious diaphoresis was established, the circulation perfectly equalized, and in no case did a single chill occur after the medicine had been used twelve hours in the intermittent, and in the remittent by the third day in the longest instance, the disease had yielded and convalescence was safely pronounced. The vertigo, tinnitus aurium, produced by the quinine frequently, is entirely avoided, and the patient enjoys a semi-somnolent state during the time of taking medicine, and at the end of twenty-four or thirty hours slowly emerges from his torpid state, his appetite slowly returns, and so far there has been no case that has had a relapse; and in fact the patient does not appreciate his illness, for he has suffered none.

These cases were all in which I tried the opiate treatment, and these were selected from their simplicity, for they were such cases as one feels safe in persevering in experimentation.

From the treatment, I should have little fears in trying it in cases of gravity; but I would recommend any one to try it in milder cases, before trying it in those of a more dangerous form. I tried merely to save quinine, and I intend to still use it, as long as I am pleased with it, in such cases as the foregoing.—*Nashville Jour. of Med. and Surg.*

